# First report on identification and genomic analysis of a novel porcine circovirus (porcine circovirus 4) in cats

**DOI:** 10.3389/fmicb.2023.1258484

**Published:** 2023-09-22

**Authors:** Tong Xu, Li-Shuang Deng, Zhi-Jie Jian, Lei Xu, Feng-Qin Li, Si-Yuan Lai, Yan-Ru Ai, Ling Zhu, Zhi-Wen Xu

**Affiliations:** ^1^College of Veterinary Medicine, Sichuan Agricultural University, Chengdu, China; ^2^College of Veterinary Medicine Sichuan Key Laboratory of Animal Epidemic Disease and Human Health, Sichuan Agricultural University, Chengdu, China

**Keywords:** porcine circovirus 4, cat, molecular detection, genetic characteristics, cross-species transmission

## Abstract

Porcine circovirus type 4 (PCV4) is an emerging circovirus, which has been detected in domestic pigs across various provinces in China and Korea. In this study, we aimed to investigate whether cats are susceptible to PCV4. For this purpose, we collected 116 cat samples from animal hospitals in Sichuan Province, China, between 2021 and 2022. Using a SYBR Green-based real-time PCR assay, we detected PCV4 in 5 out of the 116 clinical samples, indicating a positive rate of 4.31% (5/116) and confirming the presence of PCV4 in cats from Sichuan Province, China. Moreover, we successfully sequenced and analyzed the complete genome of one PCV4 strain (SCGA-Cat) along with 60 reference sequences deposited in the GenBank database. SCGA-Cat exhibited high nucleotide homology (98.2–99.0%) with PCV4 strains from other species, including dogs, pigs, dairy cows, and fur animals. Notably, the SCGA-Cat strain from cats clustered closely with a PCV4 strain derived from a pig collected in Fujian Province, China. To the best of our knowledge, this study represents the first report on the molecular detection of PCV4 in cats worldwide, which prompted us to understand the genetic diversity and cross-species transmission of the ongoing PCV4 cases. However, further investigations are needed to explore the association between PCV4 infection and clinical syndromes in cats.

## 1. Introduction

Porcine circoviruses (PCVs) belong to the Circovirus genus in the Circoviridae family and are characterized by single-stranded circular DNA viruses enclosed in an icosahedral virion with a diameter of approximately 17 nm (Opriessnig et al., 2020). Currently, at least four structurally similar PCVs have been identified, namely porcine circovirus 1 (PCV1), porcine circovirus 2 (PCV2), porcine circovirus 3 (PCV3), and porcine circovirus 4 (PCV4) (Lekcharoensuk et al., 2004; Cheung, 2012; Opriessnig et al., 2020). The genomes of PCVs are circular single-stranded DNA with sizes ranging from approximately 1.7 to 2.1 kb (Lekcharoensuk et al., 2004; Cheung, 2012; Opriessnig et al., 2020).

PCV1 was first reported in 1982 and is generally considered non-pathogenic to pigs (Tischer et al., 1974, 1986; Allan et al., 1995). In the 1990s, PCV2 was discovered and associated with a variety of clinical manifestations, including postweaning multisystemic wasting syndrome (PMWS), porcine dermatitis and nephrotic syndrome (PDNS), reproductive disorders and respiratory diseases, causing significant economic losses to the global pig industry (Nayar et al., 1997; Allan et al., 1998; Ellis et al., 1998; Kiupel et al., 1998; Morozov et al., 1998). The disease associated with PCV2 is now commonly summarized as porcine circovirus (related) disease (PCVD/PCVAD) (Nayar et al., 1997; Allan et al., 1998; Ellis et al., 1998; Kiupel et al., 1998; Morozov et al., 1998). In 2015, PCV3 was identified in tissues of pigs suffering from PDNS, reproductive failure, myocarditis or multisystemic inflammation using next-generation sequencing (NGS) methods (Phan et al., 2016; Palinski et al., 2017). Porcine circovirus 4 (PCV4), a novel species of circovirus, was first discovered in 2019 in Hunan Province of China. The virus was detected in both diseased pigs, showing severe clinical signs such as respiratory disease, enteritis, and PDNS, and healthy pigs (Zhang D. et al., 2020). Subsequently, PCV4 was reported in several provinces in China and Korea (Chen et al., 2021; Sun et al., 2021; Tian et al., 2021; Kim et al., 2022; Xu et al., 2022a). Nevertheless, given the failure to detect PCV4 DNA in pig samples from Europe (Italy and Spain) and South America (Colombia) (Franzo et al., 2020a; Vargas-Bermudez et al., 2022), it appears that the geographical distribution of PCV4 is limited. Then, PCV4 was successfully rescued from an infectious clone and proved to be pathogenic to piglets (Niu et al., 2022).

Previous studies have indicated that a variety of animals other than pigs might serve as reservoirs for PCVs. The genome of PCV2 could be identified in dogs, raccoon dogs, foxes and calves (Halami et al., 2014; Herbst and Willems, 2017; Song et al., 2019a,b). Similarly, PCV3 DNA has been detected in wild boars, dogs, cattle and mice (Zhang et al., 2018; Jiang et al., 2019; Klaumann et al., 2019; Wang et al., 2019). These findings demonstrated PCVs possesses cross-species transmission abilities and has an unexpectedly broad range and circulation in the wild. In addition, cross-species transmission of PCVs could pose a serious threat to the global pig industry and other animal industries (Turlewicz-Podbielska et al., 2022). In addition, cross-species transmission has consistently been an essential aspect of virus research. Studying cross-species transmission holds positive implications in various aspects: public health preparedness, disease control strategies, epidemiological insights, evolutionary dynamics and scientific knowledge etc. Therefore, it is crucial to give more attention to the cross-species transmission of PCVs. Since the discovery of PCV4, its genome has been identified in several non-pig animals, including dairy cattle, dogs, wild boars and fur animals (Wang et al., 2022; Wu et al., 2022; Xu et al., 2022b; Zhang et al., 2023). However, it is unclear whether other species (including cats) are involved in the spread of PCV4.

Whether cats are one of the reservoirs of PCV4, and if so, what are prevalence and genomic characteristics of PCV4 in cats? Considering the above premises, the aim of this study was to investigate the presence and circulation of PCV4 in cats. In the present study, a total 116 clinical samples were collected from Sichuan province of China, and screened for the presence of PCV4 in cats. Hoping that studying PCV4 infections in cats can provide insights into viral evolution, genetic changes, and factors influencing the spread and adaptation of viruses.

## 2. Materials and methods

### 2.1. Samples collection and DNA extraction

**Figure 1:**
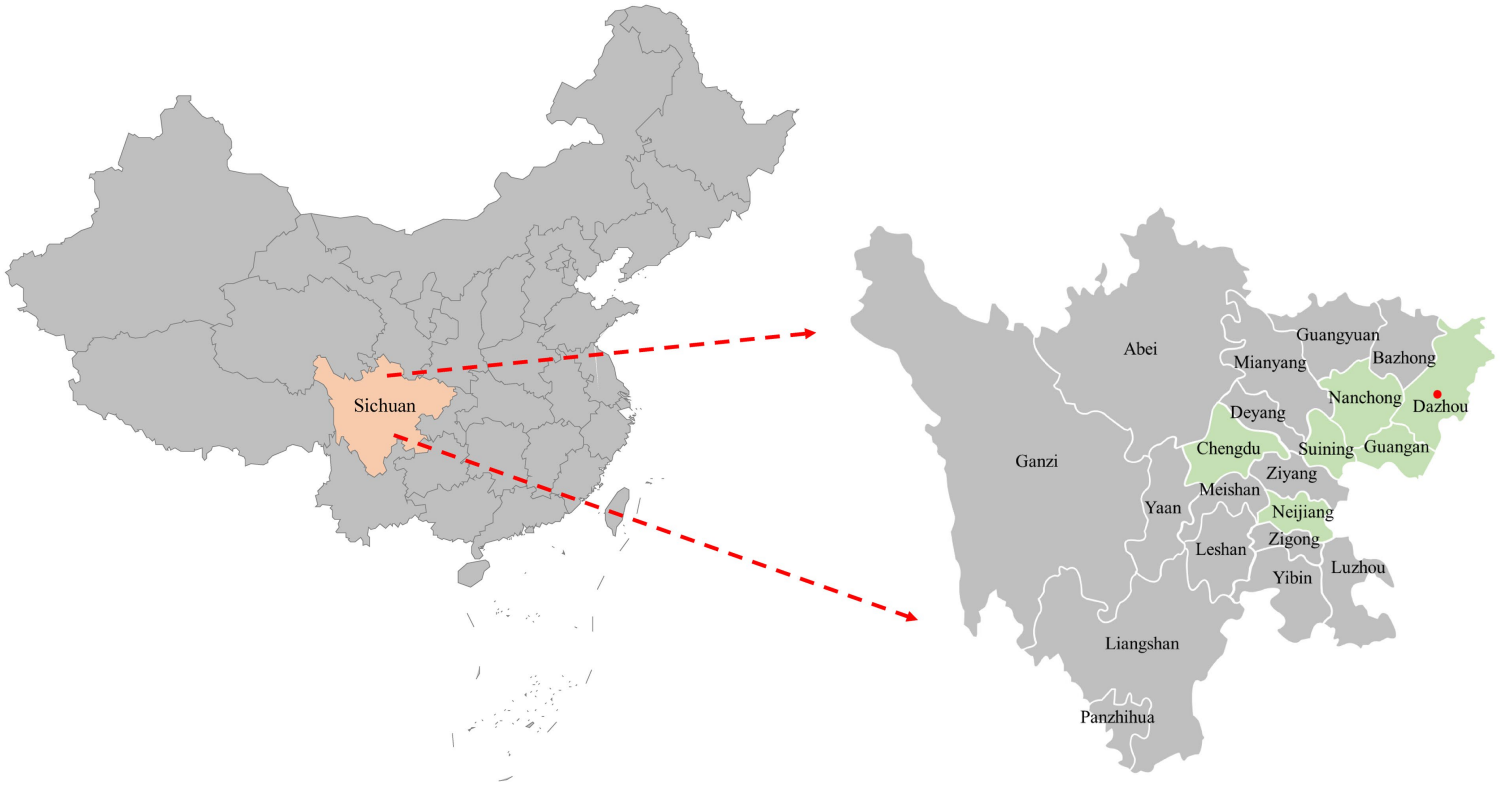
The geographical distribution of the 116 clinical samples in this study. The samples were obtained from six cities in Sichuan Province, China, represented by light green shading on the map. Cities with positive samples were indicated by red solid circles.

A total of 116 samples were randomly collected from 10 animal hospitals in 6 cities (Chengdu, Suining, Guangan, Neijiang, Nanchong and Dazhou) of Sichuan Province China during 2021–2022 (Figure 1). 87.06% (101/116) of the samples collected from diseased cats with clinical signs (including respiratory disease and enteritis) were submitted for diagnosis, while the other 12.93% (15/116) were collected from healthy animals for infectious disease surveillance. The sample types included serum, pharyngeal swabs, nasal swabs and feces.

Each sample was individually mixed with phosphate-buffered saline (PBS) and placed in a sterile 1.5 mL microcentrifuge tube containing steel beads. The mixture was homogenized at 50 Hz for 60 s at 4°C using the SCIENTZ-48 L high-throughput tissue homogenizer (Ningbo Scientz Biotechnology CO., Ltd., Ningbo, China). The homogenate underwent three cycles of freezing and thawing, followed by centrifugation at 12,000 × g. The resulting supernatant was immediately used for DNA extraction or stored at −80°C until further use. DNA extraction was performed using the FastPure Viral DNA/RNA Mini Kit (Vazyme Biotech Co., Ltd., Nanjing, China), following the manufacturer’s instructions. For the detection of PCV4 in clinical samples, a real-time PCR assay based on SYBR Green І was conducted as previously described (Xu et al., 2022a).

### 2.2. Complete genome sequencing

The complete genome of PCV4 was sequenced as described previously (Xu et al., 2022b). Three overlap primer pairs were used to sequence the complete genome and described in Supplementary Table S1. The amplified conditions of these three overlapping fragments were 98°C for 30 s; 35 cycles of 30 s at 98°C, 60°C for 30 s, and 72°C for 30 s, and a final extension for 10 min at 72°C. According to the manufacturer’s instructions, the PCR amplification products were purified using the V-ELUTE Gel Mini Purification Kit (Beijing Zoman Biotechnology Co., Ltd., Beijing, China) and cloned into the pMD18-T vector (Takara, Dalian, China) for constructing recombinant plasmid. The recombinant plasmids propagated in DH5α competent cells (Takara). The positive clones were sent to Tsingke Biotechnology Co., Ltd., Beijing, China for sequencing. The complete genome was assembled by the EditSeq and Megalign programs of the LaserGene software package (DNASTAR, Inc., Madison, WI).

### 2.3. Sequence alignment and phylogenetic analysis

Complete gene sequences of identified PCV4 strains were analyzed with all currently available 60 reference strains deposited in GenBank (accessed March 7, 2023). The information of the reference strains was shown in Supplementary Table S2.

For the analysis, nucleotide and deduced amino acid sequences were aligned using the Clustal W method of the MegAlign program. To construct the phylogenetic tree, the Molecular Evolutionary Genetics Analysis (MEGA) software (version 7.0) was utilized, applying the neighbour-joining method with a p-distance model and performing 1,000 replicates for bootstrapping.

## 3. Results

### 3.1. First detected PCV4 in cats

In this study, 116 clinical samples were collected from 10 different animal hospitals in six cities (Chengdu, Suining, Guangan, Neijiang, Nanchong and Dazhou) of Sichuan Province China during 2021–2022. PCV4 genome was found in 5 out of 116 studied clinical samples (4.31%) coming also from 2 animal hospitals out of the 10 tested animal hospitals (20%). Five positive clinical samples consisting of two feces, two serum and one nasal swab (Table 1). The information (including collection dates, geographical locations, growth stages, sample types, clinical symptoms, and co-infection status with other pathogens) of PCV4-positive cat samples in Sichuan Province from 2021 to 2022 was summarized in Table 1. The two animal hospitals with PCV4-positive samples were both from Guangan, Sichuan Province; however, none of the positive samples were detected in the other five tested cities (Chengdu, Suining, Neijiang, Nanchong and Dazhou) (Figure 1). Six other pathogens in five PCV4-positive samples had been detected in animal hospitals and were summarized in Table 1. These five pathogens included: FHV-1(feline herpesvirus-1), FCV (feline calicivirus), *M. felis*: (*Mycoplasma felis*), *C. felis* (Chlamydia felis), FCoV (feline coronavirus), FPV (feline panleukopenia virus).

**Table 1:** Origin, clinical manifestation information and case diagnostic results of PCV4 positive samples in cats in Sichuan Province, China, 2021–2022.

Sample name	Collection date	Geographical location	Animal hospital	Growth stages	Sample type	Clinical symptoms	FHV-1	FCV	*M. felis*	*C. felis*	FCoV	FPV
Sample 1	2021.09.11	Guangan	A	Kitten (0–12 months)	nasal swabs	Respiratory	+	−	+	−	−	−
Sample 2	2021.10.23	Guangan	A	Young adult (13–72 months)	serum	Respiratory	+	−	−	−	−	−
Sample 3※	2022.03.03	Guangan	A	Young adult (13–72 months)	feces	Diarrhea	−	−	−	−	−	+
Sample 4	2022.05.22	Guangan	B	Senior (>120 months)	serum	Respiratory	−	−	−	+	−	−
Sample 5※.	2022.06.12	Guangan	B	Kitten (0–12 months)	feces	Diarrhea	−	−	−	−	+	−

FHV-1, feline herpesvirus-1; FCV, feline calicivirus; *M. felis*, *Mycoplasma felis*, *C. felis*: *Chlamydia felis*; FCoV, feline coronavirus; FPV, feline panleukopenia virus. The complete genome of SCGA-Cat was acquired from sample 3 and 5 marked with ※.

### 3.2. Sequence analysis and phylogenetic studies of PCV4 strains

To further understand the genetic characteristics of PCV4 in cats, 2 PCV4 whole genomes were sequenced from two fecal samples collected from Guangan, Sichuan Province. Among which one was unique sequence (SCGA-Cat) and deposited in the GenBank under the following accession number: OQ734983.

Sequence alignment of complete genomes showed that the genome nucleotide identity between SCGA-Cat and 60 PCV4 reference strains was 98.2–99.0% (Supplementary File S1). The raw data for nucleotide homology alignments of complete genome was displayed in Supplementary File S1. The FASTA file in Supplementary File S1 is the file before the alignment. After it has been aligned in the Cluster W method of the MegAlign program, the homology data in the PDF in Supplementary File S1 can be obtained in the “Sequence Distance.” For convenience, the nucleotide homology of the complete genomes among 61 PCV4 strains in the PDF of Supplementary File S1 has been summarized in Supplementary Table S3.

**Figure 2:**
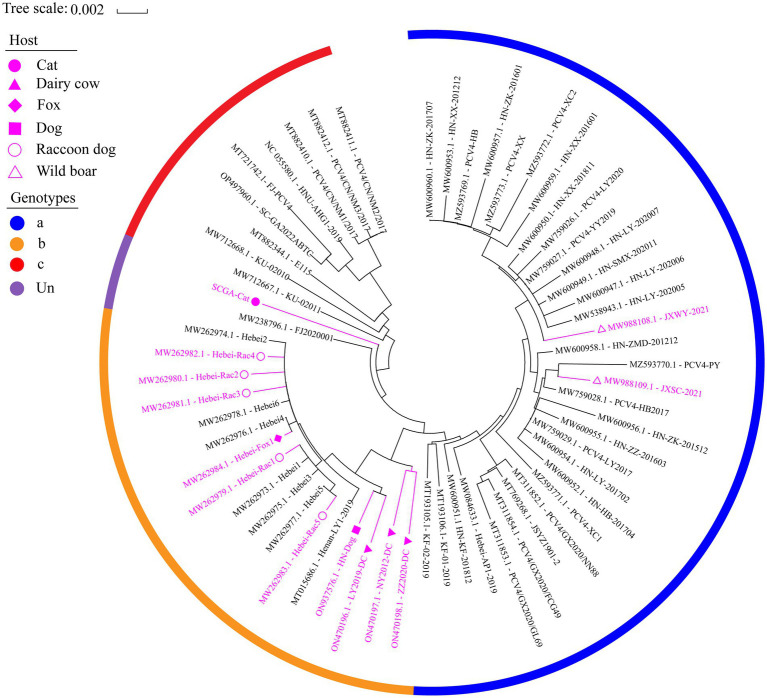
The neighbor-joining tree was constructed based on the complete genome of 61 PCV4 strains with a p-distance model and bootstrapping at 1,000 replicates. Purple color represents non-pig-derived PCV4 strains. Concisely, solid triangles, solid squares, solid diamonds, hollow circles, solid circles and hollow triangle represent PCV4 strains from dairy cows, dogs, foxes, raccoon dogs, cats and wild boars, respectively. All other unlabeled strains were from domestic pigs. The blue, orange, and red colors indicate the three genotypes (PCV4a, PCV4b and PCV4c) proposed by Xu et al. (2022a,b,c), respectively, while (undefined) is not included in these three genotypes.

Based in the proposed clade classification and amino acid marker positions described by Xu et al. (2022b), the NJ phylogenetic tree was built (Figure 2). Phylogenetic analysis showed that the 33 PCV4 strains fell into PCV4a from the four provinces (Henan, Jiangxi, Jiangsu, Guangxi, Hebei) of China (Table 2). The genomes of the PCV4a strains were detected in domestic pigs and wild boars. Five strains from Henan and 12 strains from Hebei came from five species (pig, dog, dairy cow, fox and raccoon dog) and formed a branch called PCV4b (Table 2). Six swine-derived PCV4 strains from four provinces (Sichuan, Fujian, Hunan and Neimeng) in China and three from Korea were clustered into PCV4c (Table 2). However, SCGA-Cat, along with a Fujian strain collected in 2020 (accession number, MW238796.1), was divided into an undefined cluster located between PCV4b and PCV4c. This undefined cluster was not included in the three genotypes proposed by Xu et al. (2022b).

**Table 2:** Genotypes, hosts and geographical distribution of 61 PCV4 strains.

Genotypes	Number	Geographical location	Hosts
PCV4a	33	China (Henan 26, Jiangxi 2, Jiangsu 1, Guangxi 3, Hebei 1)	Pig (31), wild boar (2)
PCV4b	17	China (Henan 5, Hebei 12)	Pig (7), dog (1) dairy cow (3), fox (1), raccoon dog (5)
PCV4c	9	China (Sichuan 1, Fujian 1, Hunan 1, Neimeng 3), Korea (3)	pig (9)
Undefined	2	China (Fujian 1, Sichuan 1※)	Pig (1), cat (1※)
Total	61	China (Henan 31, Jiangxi 2, Jiangsu 1, Guangxi 3, Hebei 13, Fujian 2, Sichuan 2, Neimeng 3, Hunan 1), Korea (3)	Pig (48), wild boar (2), dog (1), dairy cow (3), fox (1), raccoon dog (5), cat (1)

The information in parentheses after the country in the “Geographical location” column indicates the province and the number of PCV4 strains available in that province. The information in parentheses after “host” indicates the number of PCV4 strains. The complete genome of SCGA-Cat was marked with ※.

**Figure 3:**
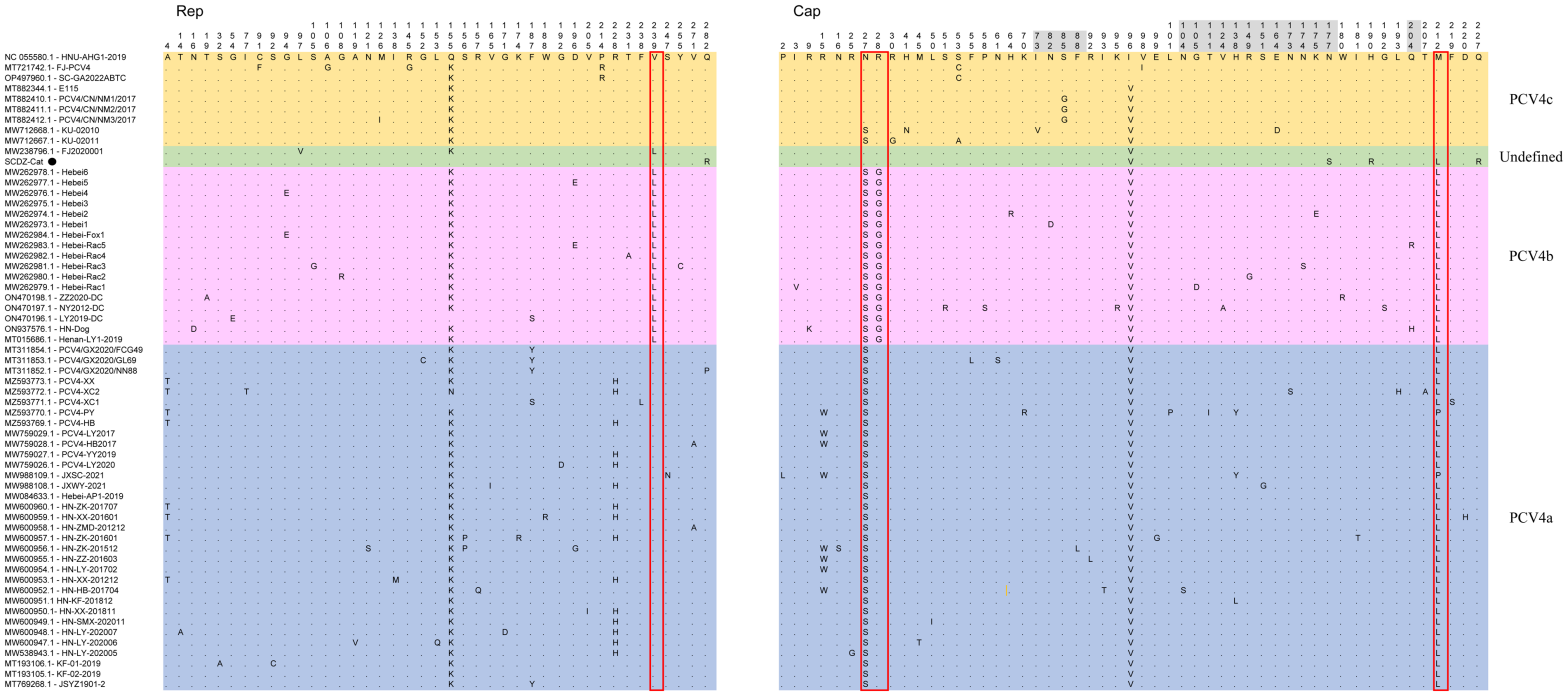
The multiple sequence alignment showed that among 61 PCV4 strains, there were 41 amino acid mutations in ORF1-encoded Rep protein and 53 in ORF2-encoded Cap protein. Light blue, light purple and light orange indicate the three genotypes (PCV4a, PCV4b and PCV4c) proposed by Xu et al. (2022a,b,c), respectively, while the strains indicated with light green are not included in these three genotypes. The red open box showed the amino acid markers of genotypes proposed by Xu et al. (2022a,b,c). The amino acid positions in the gray region are included in the potential linear B-cell epitopes predicted by Wang et al. (2021).

The nucleotides and deduced amino acid sequences of ORF2 gene of SCGA-Cat were analyzed with 60 reference strains. Among these 61 strains, 92 nucleotide mutations occurred in ORF1 and ORF2, respectively (Supplementary Figure S1), and 41 and 53 amino acid mutations occurred in ORF1-encoded Rep protein and ORF2-encoded Cap protein (Figure 3), respectively.

## 4. Discussion

Based on available data, it is evident that PCVs (PCV1-3) have a wide range of hosts and can cause clinical symptoms under field conditions, leading to significant economic losses in the pig farming and other animal industries (Turlewicz-Podbielska et al., 2022). Drawing lessons from the potentially serious threat of cross-species transmission of PCVs (PCV1-PCV3) to the global pig industry and other animal industries (Turlewicz-Podbielska et al., 2022), it is imperative to devote more attention to the cross-species transmission of the emerging porcine circovirus, PCV4. However, no information is currently available about PCV4 in cats. Therefore, a molecular epidemiological study was conducted to investigate the presence of the PCV4 genome in cats.

There are currently several studies on cross-species transmission of PCV4, with positivity rates of 19.6% (27/138) in wild boar (Wu et al., 2022), 5.99% (13/217) in dogs, 18.75% (3/16) in foxes (18/168) (Wang et al., 2022), 2.22% (26/1170) in dairy cattle (32)and 20.37% (22/108) in raccoon dogs (Wang et al., 2022). In addition, the proportion of viral genomes detected in pigs in different regions varied, with positivity rates ranging from 1.6 to 45.39% (Ha et al., 2021; Hou et al., 2022). In the present study, PCV4 DNA was identified in 5 out of 116 clinical samples (4.31%), corresponding to 2 out of 10 animal hospitals. It is very likely that different animal species involved in each study, geographical location, number of samples, the age of sampling, molecular detection methods and health status may be responsible for the different positive rates in the available reports. Two animal hospitals with PCV4-positive samples were located in Guang’an, Sichuan Province. The most likely reason for the lack of positive samples in the other five cities (Chengdu, Suining, Neijiang, Nanchong and Dazhou) might be the low number of samples screened. Meanwhile a previous study in the same city had a slightly lower rate of PCV4 positivity in swine farms, showing PDNS co-infected with PCV2 (Xu et al., 2022c). Details of the geographical distribution of the collected samples in this study are shown in Figure 1. In pigs, PCV4 possesses a wide range of histophilic (Hou et al., 2022). PCV4 DNA was detected in almost all tissues (including brain, kidney, heart, spleen) and serum of both diseased and healthy pigs (Zhang H. H. et al., 2020; Hou et al., 2022). However, there are few reports on the identification of PCV4 DNA in fluids of pigs. In this study, PCV4 DNA was identified in five different samples of the cat, including two serum, two feces and one nasal swab. Due to the limitations in the types of collected samples, the distribution of PCV4 viral load in various tissues and organs of cats could not be explored. All five positive samples were collected from sick cats, and we asked the animal hospital staffs for details about the five cats (Table 1). As depicted in Table 1, PCV4 was identified in cats concurrently infected with other pathogens but was not detected in isolation. The limited sample size may account for PCV4’s absence in healthy cats, indicating a requirement for further investigations. The five positive samples were collected from cats at different growth stages (Table 1), indicating that PCV4 is likely to infect cats of different age groups. These findings suggested that PCV4 could be detected in cats. However, there are still many questions that need to be explored, such as the relationship between PCV4 infection and pathogenicity, the distribution of the virus in various tissues post-infection, viral viremia, post-infection neutralization, and more. The isolation of the virus and animal experiments are key to further exploration. We are currently making efforts to isolate the virus, and if successful, a series of subsequent experiments will be carried out.

PCVs (PCV1-2) host jumps might be a potential threat to public health (Turlewicz-Podbielska et al., 2022). It is unclear whether PCV4 has zoonotic potential and, if so, whether cats, as human companions, play a significant role in the transmission of PCV4 between humans and animals. Moreover, studying the cross-species transmission of viruses among animals holds significant importance for scientific knowledge: advancing our understanding of cross-species transmission adds to the broader body of scientific knowledge and contributes to our understanding of viral biology and host-pathogen interactions. Thus, much attention should be paid to these potential pathways of transmission.

To understand the genetic characteristics of PCV4 in cats, the whole genome was amplified from positive samples. Two complete sequences were successfully amplified from two positive samples, while the low viral load of the remaining three positive samples precluded acquisition of their whole genomes. Interestingly, the two obtained whole genomes enjoyed 100% homology and were obtained from two different animal hospitals in Guang’an in 2022 (Table 1). The unique complete sequence (SCGA-Cat) was submitted to GenBank database with the accession number (OQ734983). Compared to the reference strains, no base insertions and deletions occurred in the whole genome of SCGA-Cat with a size of 1,170 nt. Then, SCGA-Cat was analyzed with 60 reference strains deposited in GenBank (Supplementary Table S1).

The multiple sequence alignment showed SCGA-Cat shared 98.2–99.0% whole genome nucleotide identity with 60 reference strains. The hosts of these reference strains include pigs, dogs, foxes and dairy cows (Wang et al., 2022; Wu et al., 2022; Xu et al., 2022b; Zhang et al., 2023), and the reference sequences were collected from different provinces of China and Korea from 2012 to 2022. In terms of geographic distribution, host and time, PCV-4 has high similarity (98.2–99.0%) among the available sequences, indicating a low variability of PCV4.

By consulting at the GenBank database (accessed March 7, 2023), there are only 60 PCV4 whole genome sequences in total. The limited number of whole genome sequences and the high homology between existing sequences makes it difficult to determine further genotypic classification of PCV4. Currently, there is controversy about the classification of PCV4. Despite it is too early for a definitive classification, two (PCV4a and PCV4b) or three main clades (PCV4a, PCV4b and PCV4c) have been proposed for this virus (Xu et al., 2022b). In this study, we refer to the method described by Xu et al. (2022a,b) and also classify PCV4 into three different genotypes, namely PCV4a, PCV4b and PCV4c, by combining the phylogenetic tree and amino acid sequences. SCGA-Cat was included in the phylogenetic analysis along with all currently available PCV4 whole genomes (60 in total). As is shown in Figure 2, three distinct clusters corresponding to PCV4a, PCV4b, and PCV4c genotypes were observed in the phylogenetic tree based on Xu et al.’s (2022b) proposed clade classification and amino acid marker positions. In this study, SCGA-Cat belongs to a small cluster between PCV4b and PCV4c together with a Fujian strain (accession number, MW238796.1) collected in 2020, which is not included in the three genotypes proposed by Xu et al. (2022b). When different numbers of sequences were used, the classification of some strains in this study was not consistent with the classification proposed in a previous study (Xu et al., 2022b). The specific amino acid patterns reported by Xu et al. included PCV4a (239 V in Rep, 27S, 28R and 212 L in Cap), PCV4b (239 L for Rep, 27 S, 28 G and 212 L for Cap) and PCV4c (239 V for Rep, 27 N, 28 R and 212 M for Cap). Nevertheless, in the phylogenetic tree, two strains (KU-02010 and KU-02011) belong to PCV4c, but at amino acid position 27 of Cap is S instead of N. Two strains (PCV4-PY and JXSC-2021) belong to PCV4a, but at amino acid position 212 of Cap is P instead of L. These results differ from previous studies (Xu et al., 2022b; Zhang et al., 2023), indicating the proposed classification of genotypes were not suitable. Similar to what initially occurred with PCV-2 and now occurs with PCV3 (Segalés et al., 2008; Franzo et al., 2020b), no consensus was reached and different research groups proposed independent classification criteria and protocols, leading to some confusion among veterinarians and researchers. In order to be practical and useful, PCV-4 should not be classified for its own sake, but should help to explain the underlying viral characteristics and thus contribute to the understanding of its epidemiology and potential control measures (Simmonds, 2015; Franzo et al., 2020b). Genetic distance and phylogenetic clustering should be selected as the main target criteria (Franzo et al., 2020b). Other factors, including the number of sequences within clusters, host and geographic clustering, concordance between different genomic regions, and analysis methods should also be taken into account to produce a classification that can be used effectively for research and diagnosis (Franzo et al., 2020b). With this in mind, we encourage more researchers to share more whole genome sequences with annotation in a free database for more precise and meaningful typing schemes.

Among 61 PCV4 strains, there were 41 amino acid mutations in Rep protein and 53 in Cap protein (Figure 3). In comparison with the first identified PCV4 strain (HNU-AHG1-2019) derived from pigs, no amino acid insertions or deletions were observed on the Rep and Cap of SCGA, but one amino acid mutation (Q282R) and three amino acid mutations (I96V, N177S, H190R, M212L and Q227R) were found on the Rep and Cap (Figure 3), respectively. Notably, Q282R of the Rep protein and N177S, H190R and Q227R of the Cap protein are unique in SCGA-Cat compared to the other 60 available PCV4 reference sequences. We speculate that these unique amino acids might be a result of the evolution of PCV4 towards a more adapted host (cat). Whether these unique mutations in the SCGA strain are related to the adaptation of PCV4 to the host is warranted for further investigation. SCGA-Cat also contained the essential elements for the replication of circoviruses in pig-origin PCV4 strains predicted by Nguyen et al. (Zhang et al., 2023), such as the origin of DNA replication, endonuclease and helicase.

The nuclear localization signal (NLS) region is an arginine-rich region within the genus circovirus that mediates the nuclear localization of the viral genome (Liu et al., 2001; Shuai et al., 2008; Mou et al., 2019). Recently, it was reported that the NLS of PCV4 Cap is located at the N-terminal residues 1–20 (Zhou et al., 2021), which was also observed in SCDA-Cat. In the PCV4 Cap, five potential linear B cell epitopes with high antigenicity were predicted by Wang et al. (2021), including epitope A: 72–88, 104–112, epitope B: 122–177, epitope C:199–205, and epitope D:219–225 (Figure 3). Of the 53 amino acid bursts in the Cap of the 61 PCV4 strains, 17 are located in the predicted epitope region and may lead to altered antigenicity of the Cap protein, which requires further study.

## 5. Conclusion

Overall, to our knowledge, this study is the first to identify the genome of PCV4 in clinical samples from cats, indicating that cats may be one of the hosts of PCV4. In addition, the first complete genome sequence (SCGA-Cat) of PCV4 in cat was amplified to explore the genetic characteristics of PCV4 in cats. These findings will further enrich our understanding of this newly emerging circovirus.

## Data availability statement

The datasets presented in this study can be found in online repositories. The names of the repository/repositories and accession number(s) can be found in the article/Supplementary material.

## Ethics statement

All experimental procedures were reviewed and approved by the Sichuan Agriculture University Animal Care and Use Committee (license number SCXK (Sichuan) 2013–0001). The study was conducted in accordance with the local legislation and institutional requirements.

## Author contributions

TX: Conceptualization, Data curation, Formal analysis, Writing – original draft. L-SD: Formal analysis, Writing – review & editing. Z-JJ: Formal analysis, Writing – review & editing. LX: Software, Writing – review & editing. F-QL: Software, Writing – review & editing. S-YL: Writing – review & editing. Y-RA: Supervision, Writing – review & editing. LZ: Conceptualization, Funding acquisition, Project administration, Writing – review & editing. Z-WX: Funding acquisition, Project administration, Writing – review & editing.
