# Supplementary material for: First report on identification and genomic analysis of a novel porcine circovirus (porcine circovirus 4) in cats
Source: Front Microbiol. 2023 Sep 22;14:1258484. doi: 10.3389/fmicb.2023.1258484 (PMC10556453; doi:10.3389/fmicb.2023.1258484)
Supplement: Supplementary file 1 [file Data_Sheet_1.zip › Table S1.DOCX]

Supplementary Table 1 List of primer sequences used in this study

| Primer name | Nucleotide sequence (5’-3’) | Primer locations (bp) | Product size | |
| --- | --- | --- | --- | --- |
| PCV4-1F | GAGGTTCCACCCGTTTAAG | 260-278 | 577 |  |
| PCV4-1R | CCAGTCCTTGATCTGCTTGTTG | 815-836 |  |  |
| PCV4-2F | GCCAAGACAATGTGGATTACC | 792-812 | 690 |  |
| PCV4-2R | AGCCTCCCATTTGCATATTACC | 1460-1481 |  |  |
| PCV4-3F | CCACATAGTCTCCATCCAGTTG | 1361-1382 | 769 |  |
| PCV4-3R | CCCTCCTTTGGAGCAATACTT | 339-359 |  |  |
