# Supplementary figures and images for: First report on identification and genomic analysis of a novel porcine circovirus (porcine circovirus 4) in cats
Source: Front Microbiol. 2023 Sep 22;14:1258484. doi: 10.3389/fmicb.2023.1258484 (PMC10556453; doi:10.3389/fmicb.2023.1258484)

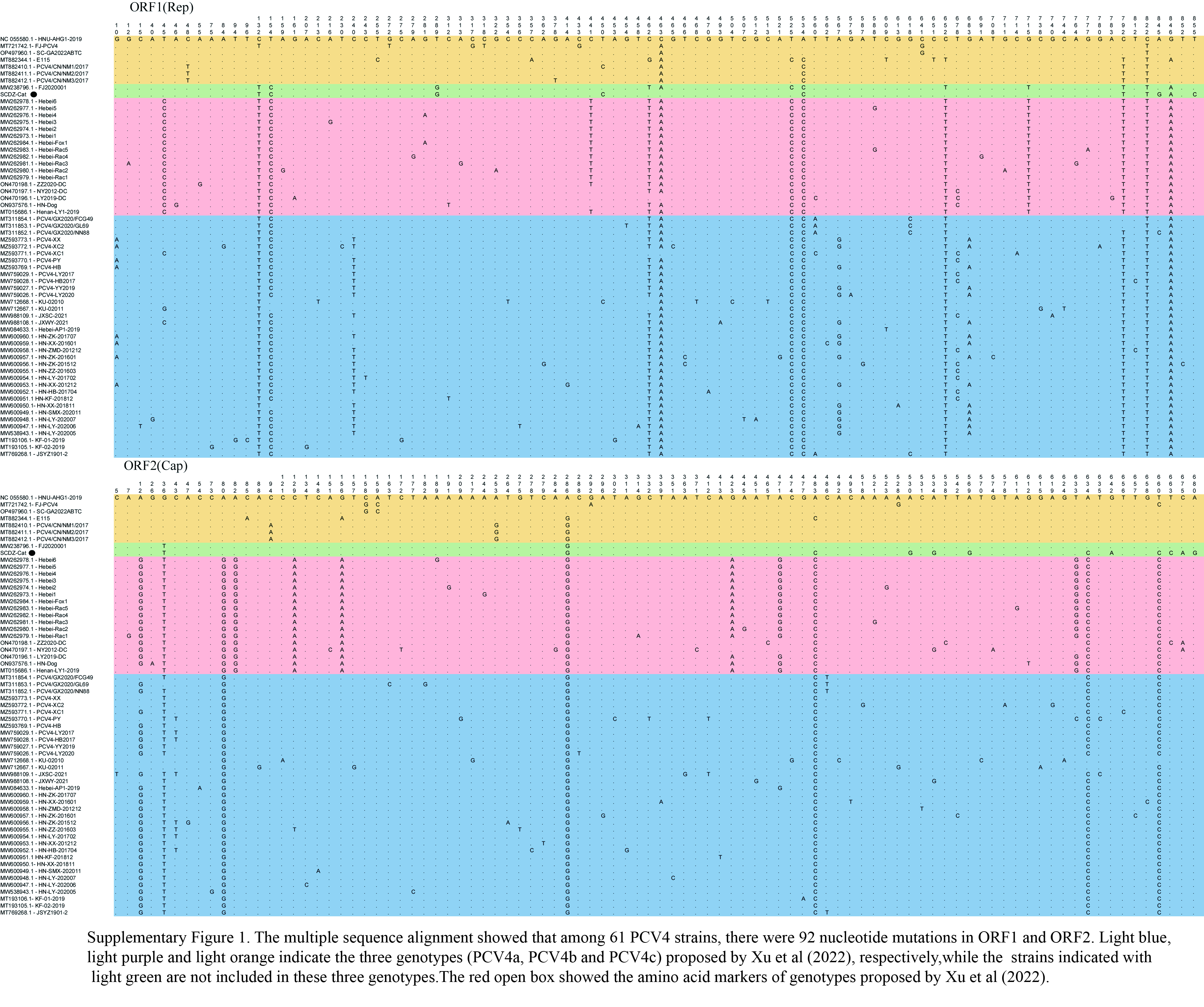

Supplement: Supplementary file 1 [file Data_Sheet_1.zip › Figure S1.JPEG]
