# Supplementary material for: First report on identification and genomic analysis of a novel porcine circovirus (porcine circovirus 4) in cats
Source: Front Microbiol. 2023 Sep 22;14:1258484. doi: 10.3389/fmicb.2023.1258484 (PMC10556453; doi:10.3389/fmicb.2023.1258484)
Supplement: Supplementary file 1 [file Data_Sheet_1.zip › Supplementary File 1.ZIP!/Supplementary 2.pdf]

Percent Identity

Divergence

	1	2	3	4	5	6	7	8	9	10	11	12	13	14	15	16	17	18	19	20	21	22	23	24	25	26	27	28	29	30	31	32	33	34	35	36	37	38	39	40	41	42	43	44	45	46	47	48	49	50
1		99.2	99.2	99.3	99.2	99.0	99.3	99.0	99.2	99.0	99.0	98.8	98.9	99.2	98.8	99.1	98.8	99.2	99.1	99.3	99.2	99.3	99.1	98.9	99.0	99.0	99.2	99.0	98.9	99.0	98.9	98.9	99.0	99.1	99.0	99.1	99.0	99.0	99.0	99.4	99.2	99.0	99.3	98.8	98.9	99.2	99.3	99.0	99.1	99.4
2	0.9		99.5	99.5	99.5	98.9	98.8	98.6	98.6	98.2	98.2	98.3	98.2	98.6	98.2	98.7	98.2	98.5	99.7	98.7	98.6	98.7	98.5	98.7	98.9	98.4	98.5	98.3	98.2	98.3	98.2	98.2	98.2	98.4	98.3	98.4	98.3	98.3	98.3	98.8	98.5	98.4	98.6	98.2	98.3	98.5	98.6	98.4	98.5	98.8
3	0.8	0.5		99.9	99.9	99.0	98.8	98.5	98.6	98.3	98.3	98.2	98.2	98.6	98.2	98.6	98.2	98.6	99.4	98.8	98.6	98.8	98.5	98.8	98.9	98.5	98.6	98.4	98.2	98.4	98.2	98.2	98.3	98.4	98.4	98.4	98.4	98.4	98.4	98.8	98.6	98.4	98.7	98.2	98.4	98.6	98.7	98.4	98.5	98.8
4	0.7	0.5	0.1		99.9	99.0	98.8	98.6	98.7	98.4	98.4	98.3	98.3	98.7	98.3	98.7	98.3	98.6	99.5	98.8	98.7	98.8	98.6	98.8	99.0	98.5	98.6	98.4	98.3	98.4	98.3	98.3	98.4	98.5	98.4	98.5	98.4	98.4	98.4	98.9	98.6	98.5	98.8	98.3	98.4	98.6	98.8	98.5	98.6	98.9
5	0.8	0.5	0.1	0.1		99.0	98.8	98.5	98.6	98.3	98.3	98.2	98.2	98.6	98.2	98.6	98.2	98.6	99.4	98.8	98.6	98.8	98.5	98.9	98.9	98.5	98.6	98.4	98.2	98.4	98.2	98.2	98.3	98.4	98.4	98.4	98.4	98.4	98.4	98.8	98.6	98.4	98.7	98.2	98.4	98.6	98.7	98.4	98.5	98.8
6	1.0	1.1	1.0	1.0	1.0		98.7	98.5	98.6	98.4	98.4	98.3	98.4	98.6	98.2	98.5	98.2	98.5	98.9	98.7	98.6	98.7	98.5	98.6	98.8	98.4	98.5	98.5	98.4	98.5	98.4	98.4	98.4	98.5	98.5	98.5	98.5	98.5	98.5	98.9	98.5	98.4	98.6	98.2	98.3	98.5	98.6	98.4	98.5	98.8
7	0.7	1.2	1.3	1.2	1.3	1.3		99.7	99.8	98.9	98.9	98.8	98.8	99.3	98.9	99.3	98.9	99.3	98.8	99.4	99.3	99.4	99.2	98.6	98.9	99.2	99.3	98.9	98.8	98.9	98.8	98.8	98.9	99.0	98.9	99.0	98.9	98.9	98.9	99.5	99.3	99.1	99.4	98.9	99.0	99.3	99.4	99.1	99.2	99.4
8	1.0	1.4	1.5	1.4	1.5	1.5	0.3		99.7	98.6	98.6	98.5	98.6	99.1	98.7	99.2	98.7	99.2	98.5	99.3	99.2	99.3	99.1	98.4	98.7	99.0	99.0	98.7	98.6	98.7	98.6	98.6	98.6	98.8	98.7	98.8	98.7	98.7	98.7	99.3	99.2	99.0	99.3	98.8	98.9	99.2	99.3	99.0	99.1	99.2
9	0.8	1.4	1.4	1.3	1.4	1.4	0.2	0.3		98.9	98.9	98.8	98.8	99.3	98.9	99.4	98.9	99.4	98.5	99.5	99.4	99.5	99.3	98.5	98.8	99.3	99.3	98.9	98.8	98.9	98.8	98.8	98.9	99.0	98.9	99.0	98.9	98.9	98.9	99.5	99.4	99.2	99.5	99.0	99.2	99.4	99.5	99.2	99.3	99.4
10	1.0	1.8	1.7	1.7	1.7	1.7	1.1	1.4	1.1		99.2	98.9	99.0	98.8	98.4	98.9	98.4	98.8	98.2	99.0	98.9	99.0	98.8	98.2	98.5	98.7	98.8	99.3	99.2	99.3	99.2	99.2	99.2	99.3	99.3	99.3	99.3	99.3	99.3	99.0	98.8	98.6	98.9	98.5	98.6	98.9	98.9	98.6	98.8	99.0
11	1.0	1.8	1.7	1.7	1.7	1.7	1.1	1.4	1.1	0.8		99.0	99.2	98.8	98.4	99.0	98.5	98.8	98.2	99.1	99.0	99.0	98.8	98.2	98.5	98.8	98.9	99.2	99.0	99.2	99.0	99.0	99.1	99.2	99.2	99.2	99.2	99.2	99.2	99.0	98.8	98.6	99.0	98.5	98.7	99.0	99.0	98.6	98.9	99.2
12	1.2	1.7	1.8	1.7	1.8	1.7	1.3	1.5	1.3	1.1	1.0		99.4	98.6	98.2	99.2	98.4	98.6	98.1	98.9	98.8	98.8	98.6	98.1	98.5	98.6	98.6	99.4	99.3	99.4	99.3	99.3	99.4	99.5	99.4	99.5	99.4	99.4	99.4	99.0	98.6	98.5	98.9	98.3	98.5	98.8	98.9	98.5	98.7	99.0
13	1.1	1.8	1.8	1.7	1.8	1.7	1.2	1.4	1.2	1.0	0.9	0.6		98.7	98.3	98.9	98.5	98.8	98.1	99.0	98.9	98.9	98.7	98.1	98.5	98.8	98.8	99.5	99.4	99.5	99.4	99.4	99.5	99.6	99.5	99.6	99.5	99.5	99.5	99.1	98.8	98.6	99.0	98.4	98.6	98.9	99.0	98.6	98.8	99.2
14	0.8	1.4	1.4	1.3	1.4	1.4	0.7	0.9	0.7	1.3	1.3	1.4	1.3		99.6	99.2	99.4	99.9	98.5	99.5	99.5	99.8	99.5	98.5	98.8	99.3	99.5	98.8	98.7	98.8	98.7	98.7	98.8	98.9	98.8	98.9	98.8	98.8	98.8	99.4	99.9	99.7	99.5	99.6	99.2	99.4	99.5	99.8	99.3	99.4
15	1.2	1.8	1.8	1.7	1.8	1.8	1.1	1.3	1.1	1.7	1.7	1.8	1.7	0.4		98.8	99.0	99.5	98.1	99.2	99.2	99.4	99.2	98.1	98.4	98.9	99.1	98.4	98.3	98.4	98.3	98.3	98.4	98.5	98.4	98.5	98.4	98.4	98.4	99.0	99.5	99.3	99.1	99.2	98.8	99.0	99.1	99.4	98.9	99.0
16	0.9	1.3	1.4	1.3	1.4	1.5	0.7	0.9	0.6	1.1	1.0	0.9	1.1	0.8	1.2		98.9	99.3	98.5	99.5	99.4	99.4	99.2	98.5	98.9	99.3	99.3	98.9	98.8	98.9	98.8	98.8	98.9	99.0	98.9	99.0	98.9	98.9	98.9	99.4	99.3	99.1	99.5	98.9	99.2	99.4	99.5	99.1	99.3	99.4
17	1.2	1.8	1.8	1.7	1.8	1.8	1.1	1.3	1.1	1.7	1.5	1.7	1.5	0.6	1.0	1.1		99.3	98.1	99.4	99.4	99.2	98.9	98.1	98.4	99.3	98.9	98.5	98.4	98.5	98.4	98.4	98.4	98.5	98.5	98.5	98.5	98.5	98.5	99.0	99.3	99.0	99.2	99.0	99.0	99.2	99.3	99.2	99.2	99.1
18	0.9	1.5	1.4	1.4	1.4	1.5	0.7	0.9	0.6	1.2	1.2	1.4	1.3	0.1	0.5	0.7	0.7		98.5	99.6	99.6	99.8	99.6	98.5	98.8	99.3	99.4	98.9	98.8	98.9	98.8	98.8	98.8	98.9	98.8	98.9	98.9	98.9	98.9	99.4	100.0	99.7	99.5	99.7	99.2	99.4	99.5	99.8	99.4	99.4
19	0.9	0.3	0.6	0.5	0.6	1.1	1.3	1.5	1.5	1.8	1.8	1.9	1.9	1.5	1.9	1.5	1.9	1.5		98.6	98.5	98.6	98.4	98.6	98.8	98.4	98.5	98.2	98.1	98.2	98.1	98.1	98.2	98.3	98.2	98.3	98.2	98.2	98.2	98.7	98.5	98.3	98.6	98.1	98.2	98.5	98.6	98.3	98.4	98.7
20	0.7	1.3	1.3	1.2	1.3	1.3	0.6	0.7	0.5	1.0	0.9	1.1	1.0	0.5	0.9	0.5	0.6	0.4	1.4		99.9	99.8	99.5	98.6	98.9	99.7	99.4	99.0	98.9	99.0	98.9	98.9	99.0	99.1	99.0	99.1	99.0	99.0	99.0	99.6	99.6	99.3	99.8	99.3	99.6	99.8	99.9	99.4	99.8	99.6
21	0.8	1.4	1.4	1.3	1.4	1.4	0.7	0.8	0.6	1.1	1.0	1.2	1.1	0.5	0.9	0.6	0.6	0.4	1.5	0.1		99.7	99.4	98.5	98.8	99.6	99.3	98.9	98.8	98.9	98.8	98.8	98.9	99.0	98.9	99.0	98.9	98.9	98.9	99.5	99.6	99.3	99.8	99.3	99.5	99.7	99.8	99.4	99.7	99.6
22	0.7	1.3	1.3	1.2	1.3	1.3	0.6	0.7	0.5	1.0	1.0	1.2	1.1	0.2	0.6	0.6	0.9	0.2	1.4	0.2	0.3		99.8	98.6	98.9	99.5	99.6	99.0	98.9	99.0	98.9	98.9	99.0	99.1	99.0	99.1	99.0	99.0	99.0	99.6	99.8	99.5	99.7	99.5	99.4	99.6	99.7	99.7	99.5	99.5
23	0.9	1.5	1.5	1.4	1.5	1.5	0.8	0.9	0.7	1.3	1.3	1.4	1.3	0.5	0.9	0.8	1.1	0.4	1.6	0.5	0.6	0.2		98.4	98.7	99.3	99.4	98.8	98.8	98.8	98.7	98.7	98.9	98.9	98.9	98.9	98.9	98.8	98.9	99.4	99.6	99.3	99.5	99.3	99.2	99.4	99.5	99.4	99.3	99.3
24	1.1	1.3	1.3	1.2	1.1	1.4	1.4	1.6	1.5	1.8	1.8	1.9	1.9	1.5	1.9	1.5	1.9	1.5	1.4	1.4	1.5	1.4	1.6		98.8	98.4	98.5	98.2	98.1	98.2	98.1	98.1	98.2	98.3	98.2	98.3	98.2	98.2	98.2	98.7	98.5	98.3	98.6	98.1	98.2	98.5	98.6	98.3	98.4	98.7
25	1.0	1.1	1.1	1.0	1.1	1.2	1.1	1.3	1.2	1.5	1.5	1.5	1.5	1.2	1.6	1.1	1.6	1.3	1.2	1.1	1.2	1.1	1.3	1.2		98.6	98.8	98.6	98.5	98.6	98.5	98.5	98.5	98.6	98.6	98.6	98.6	98.6	98.6	99.0	98.8	98.6	98.9	98.4	98.5	98.8	98.9	98.6	98.7	99.0
26	1.0	1.6	1.5	1.5	1.5	1.6	0.9	1.0	0.7	1.3	1.2	1.4	1.3	0.7	1.1	0.7	0.7	0.7	1.7	0.3	0.4	0.5	0.7	1.7	1.4		99.1	98.8	98.6	98.8	98.6	98.6	98.7	98.8	98.8	98.8	98.8	98.8	98.8	99.3	99.3	99.0	99.5	99.0	99.3	99.5	99.7	99.2	99.5	99.3
27	0.9	1.5	1.4	1.4	1.4	1.5	0.7	1.0	0.7	1.2	1.1	1.4	1.3	0.5	0.9	0.7	1.1	0.6	1.5	0.6	0.7	0.4	0.6	1.5	1.2	0.9		98.9	98.8	98.9	98.8	98.8	98.8	98.9	98.9	98.9	98.9	98.9	98.9	99.3	99.4	99.3	99.3	99.1	99.0	99.2	99.3	99.3	99.2	99.2
28	1.0	1.7	1.7	1.6	1.7	1.5	1.1	1.3	1.1	0.7	0.9	0.6	0.5	1.2	1.6	1.1	1.5	1.1	1.8	1.0	1.1	1.0	1.2	1.8	1.4	1.3	1.1		99.7	99.8	99.7	99.7	99.7	99.8	99.8	99.9	99.8	99.8	99.8	99.2	98.9	98.7	99.0	98.5	98.6	98.9	99.0	98.7	98.8	99.1
29	1.1	1.8	1.8	1.7	1.8	1.7	1.2	1.4	1.2	0.9	1.0	0.7	0.6	1.3	1.7	1.2	1.7	1.3	1.9	1.1	1.2	1.1	1.2	1.9	1.5	1.4	1.3	0.3		99.7	99.5	99.5	99.7	99.7	99.9	99.7	99.8	99.7	99.8	99.1	98.8	98.6	98.9	98.4	98.5	98.8	98.9	98.6	98.7	99.0
30	1.0	1.7	1.7	1.6	1.7	1.5	1.1	1.3	1.1	0.7	0.9	0.6	0.5	1.2	1.6	1.1	1.5	1.1	1.8	1.0	1.1	1.0	1.2	1.8	1.4	1.3	1.1	0.2	0.3		99.7	99.7	99.7	99.8	99.8	99.8	99.8	99.8	99.8	99.2	98.9	98.7	99.0	98.5	98.6	98.9	99.0	98.7	98.8	99.1
31	1.1	1.8	1.8	1.7	1.8	1.7	1.2	1.4	1.2	0.9	1.0	0.7	0.6	1.3	1.7	1.2	1.7	1.3	1.9	1.1	1.2	1.1	1.3	1.9	1.5	1.4	1.3	0.3	0.5	0.3		99.5	99.6	99.7	99.7	99.7	99.7	99.7	99.7	99.1	98.8	98.6	98.9	98.4	98.5	98.8	98.9	98.6	98.7	99.0
32	1.1	1.8	1.8	1.7	1.8	1.7	1.2	1.4	1.2	0.9	1.0	0.7	0.6	1.3	1.7	1.2	1.7	1.3	1.9	1.1	1.2	1.1	1.3	1.9	1.5	1.4	1.3	0.3	0.5	0.3	0.5		99.6	99.7	99.7	99.7	99.7	99.7	99.7	99.1	98.8	98.6	98.9	98.4	98.5	98.8	98.9	98.6	98.7	99.0
33	1.0	1.8	1.7	1.7	1.7	1.6	1.1	1.4	1.1	0.8	0.9	0.6	0.5	1.3	1.7	1.1	1.6	1.2	1.8	1.0	1.1	1.0	1.1	1.8	1.5	1.3	1.2	0.3	0.3	0.3	0.4	0.4		99.8	99.8	99.8	99.8	99.7	99.8	99.2	98.8	98.6	98.9	98.5	98.6	98.9	98.9	98.6	98.8	99.0
34	0.9	1.7	1.6	1.5	1.6	1.5	1.0	1.3	1.0	0.7	0.8	0.5	0.4	1.1	1.5	1.0	1.5	1.1	1.7	0.9	1.0	0.9	1.1	1.7	1.4	1.2	1.1	0.2	0.3	0.2	0.3	0.3	0.2		99.8	99.9	99.8	99.8	99.8	99.3	98.9	98.8	99.0	98.6	98.7	99.0	99.0	98.8	98.9	99.2
35	1.0	1.7	1.7	1.6	1.7	1.5	1.1	1.3	1.1	0.7	0.9	0.6	0.5	1.2	1.6	1.1	1.5	1.1	1.8	1.0	1.1	1.0	1.1	1.8	1.4	1.3	1.1	0.2	0.1	0.2	0.3	0.3	0.2	0.2		99.8	99.9	99.8	99.9	99.2	98.9	98.7	99.0	98.5	98.6	98.9	99.0	98.7	98.8	99.1
36	0.9	1.7	1.6	1.5	1.6	1.5	1.0	1.3	1.0	0.7	0.8	0.5	0.4	1.1	1.5	1.0	1.5	1.1	1.7	0.9	1.0	0.9	1.1	1.7	1.4	1.2	1.1	0.1	0.3	0.2	0.3	0.3	0.2	0.1	0.2		99.8	99.8	99.8	99.3	98.9	98.8	99.0	98.6	98.7	99.0	99.0	98.8	98.9	99.2
37	1.0	1.7	1.7	1.6	1.7	1.5	1.1	1.3	1.1	0.7	0.9	0.6	0.5	1.2	1.6	1.1	1.5	1.1	1.8	1.0	1.1	1.0	1.1	1.8	1.4	1.3	1.1	0.2	0.2	0.2	0.3	0.3	0.2	0.2	0.1	0.2		99.8	99.9	99.2	98.9	98.7	99.0	98.5	98.6	98.9	99.0	98.7	98.8	99.1
38	1.0	1.7	1.7	1.6	1.7	1.5	1.1	1.3	1.1	0.7	0.9	0.6	0.5	1.2	1.6	1.1	1.5	1.1	1.8	1.0	1.1	1.0	1.2	1.8	1.4	1.3	1.1	0.2	0.3	0.2	0.3	0.3	0.3	0.2	0.2	0.2	0.2		99.8	99.2	98.9	98.7	99.0	98.5	98.6	98.9	99.0	98.7	98.8	99.1
39	1.0	1.7	1.7	1.6	1.7	1.5	1.1	1.3	1.1	0.7	0.9	0.6	0.5	1.2	1.6	1.1	1.5	1.1	1.8	1.0	1.1	1.0	1.1	1.8	1.4	1.3	1.1	0.2	0.2	0.2	0.3	0.3	0.2	0.2	0.1	0.2	0.1	0.2		99.2	98.9	98.7	99.0	98.5	98.6	98.9	99.0	98.7	98.8	99.1
40	0.6	1.3	1.2	1.1	1.2	1.1	0.5	0.7	0.5	1.0	1.0	1.0	0.9	0.6	1.0	0.6	1.0	0.6	1.3	0.4	0.5	0.4	0.6	1.3	1.0	0.7	0.7	0.8	0.9	0.8	0.9	0.9	0.9	0.7	0.8	0.7	0.8	0.8	0.8		99.4	99.3	99.5	99.1	99.2	99.4	99.5	99.3	99.4	99.5
41	0.9	1.5	1.4	1.4	1.4	1.5	0.7	0.9	0.6	1.2	1.2	1.4	1.3	0.1	0.5	0.7	0.7	0.0	1.5	0.4	0.4	0.2	0.4	1.5	1.3	0.7	0.6	1.1	1.3	1.1	1.3	1.3	1.2	1.1	1.1	1.1	1.1	1.1	1.1	0.6		99.7	99.5	99.7	99.2	99.4	99.5	99.8	99.4	99.4
42	1.0	1.7	1.6	1.5	1.6	1.7	0.9	1.0	0.8	1.4	1.4	1.5	1.4	0.3	0.7	0.9	1.0	0.3	1.7	0.7	0.7	0.5	0.7	1.7	1.4	1.0	0.7	1.3	1.4	1.3	1.4	1.4	1.4	1.3	1.3	1.3	1.3	1.3	1.3	0.7	0.3		99.3	99.4	98.9	99.2	99.3	99.5	99.1	99.3
43	0.7	1.4	1.3	1.3	1.3	1.4	0.6	0.7	0.5	1.1	1.0	1.1	1.0	0.5	0.9	0.5	0.8	0.5	1.4	0.2	0.2	0.3	0.5	1.4	1.1	0.5	0.7	1.0	1.1	1.0	1.1	1.1	1.1	1.0	1.0	1.0	1.0	1.0	1.0	0.5	0.5	0.7		99.2	99.4	99.7	99.8	99.4	99.6	99.5
44	1.2	1.8	1.8	1.7	1.8	1.8	1.1	1.2	1.0	1.5	1.5	1.7	1.6	0.4	0.8	1.1	1.0	0.3	1.9	0.7	0.7	0.5	0.7	1.9	1.6	1.0	0.9	1.5	1.6	1.5	1.6	1.6	1.5	1.4	1.5	1.4	1.5	1.5	1.5	0.9	0.3	0.6	0.8		99.0	99.1	99.2	99.5	99.0	99.1
45	1.1	1.7	1.7	1.6	1.7	1.7	1.0	1.1	0.9	1.4	1.3	1.5	1.4	0.9	1.3	0.9	1.0	0.8	1.8	0.4	0.5	0.6	0.9	1.8	1.5	0.7	1.0	1.4	1.5	1.4	1.5	1.5	1.4	1.3	1.4	1.3	1.4	1.4	1.4	0.8	0.8	1.1	0.6	1.0		99.4	99.5	99.0	99.4	99.2
46	0.9	1.5	1.4	1.4	1.4	1.5	0.7	0.9	0.6	1.1	1.0	1.2	1.1	0.6	1.0	0.6	0.8	0.6	1.5	0.2	0.3	0.4	0.6	1.5	1.3	0.5	0.8	1.1	1.2	1.1	1.2	1.2	1.1	1.0	1.1	1.0	1.1	1.1	1.1	0.6	0.6	0.9	0.3	0.9	0.6		99.8	99.3	99.6	99.4
47	0.7	1.4	1.3	1.3	1.3	1.4	0.6	0.7	0.5	1.1	1.0	1.1	1.0	0.5	0.9	0.5	0.7	0.5	1.4	0.1	0.2	0.3	0.5	1.4	1.1	0.3	0.7	1.0	1.1	1.0	1.1	1.1	1.1	1.0	1.0	1.0	1.0	1.0	1.0	0.5	0.5	0.7	0.2	0.8	0.5	0.2		99.4	99.7	99.5
48	1.0	1.7	1.6	1.5	1.6	1.7	0.9	1.0	0.8	1.4	1.4	1.5	1.4	0.2	0.6	0.9	0.9	0.2	1.7	0.6	0.6	0.3	0.6	1.7	1.4	0.9	0.7	1.3	1.4	1.3	1.4	1.4	1.4	1.3	1.3	1.3	1.3	1.3	1.3	0.7	0.2	0.5	0.6	0.5	1.0	0.7	0.6		99.2	99.3
49	0.9	1.5	1.5	1.4	1.5	1.5	0.8	0.9	0.7	1.3	1.1	1.3	1.2	0.7	1.1	0.7	0.9	0.6	1.6	0.2	0.3	0.5	0.7	1.6	1.3	0.5	0.9	1.2	1.3	1.2	1.3	1.3	1.3	1.1	1.2	1.1	1.2	1.2	1.2	0.6	0.6	0.9	0.4	1.0	0.6	0.4	0.3	0.8		99.4
50	0.6	1.3	1.2	1.1	1.2	1.3	0.6	0.9	0.6	1.0	0.9	1.0	0.8	0.6	1.0	0.6	0.9	0.6	1.3	0.4	0.4	0.5	0.7	1.3	1.0	0.7	0.8	0.9	1.0	0.9	1.0	1.0	1.0	0.9	0.9	0.9	0.9	0.9	0.9	0.5	0.6	0.7	0.5	0.9	0.8	0.6	0.5	0.7	0.6	
51	0.8	1.3	1.3	1.2	1.3	1.3	0.7	0.8	0.6	1.1	1.1	1.3	1.2	0.3	0.7	0.7	1.0	0.3	1.4	0.3	0.5	0.1	0.3	1.4	1.1	0.6	0.5	1.1	1.2	1.1	1.2	1.2	1.1	1.0	1.1	1.0	1.1	1.1	1.1	0.5	0.3	0.6	0.4	0.6	0.7	0.5	0.4	0.5	0.6	0.6
52	0.7	1.4	1.3	1.3	1.3	1.4	0.6	0.7	0.5	1.1	1.1	1.3	1.1	0.3	0.7	0.6	0.9	0.2	1.4	0.3	0.4	0.1	0.3	1.4	1.1	0.6	0.5	1.0	1.1	1.0	1.1	1.1	1.1	1.0	1.0	1.0	1.0	1.0	1.0	0.5	0.2	0.5	0.3	0.6	0.7	0.5	0.3	0.4	0.5	0.6
53	0.9	1.5	1.5	1.4	1.5	1.5	0.8	0.9	0.7	1.3	1.3	1.4	1.3	0.5	0.9	0.8	1.1	0.4	1.6	0.5	0.6	0.2	0.5	1.6	1.3	0.7	0.6	1.2	1.3	1.2	1.3	1.3	1.3	1.1	1.2	1.1	1.2	1.2	1.2	0.6	0.4	0.7	0.5	0.7	0.9	0.6	0.5	0.6	0.7	0.7
54	0.9	1.5	1.5	1.4	1.5	1.5	0.8	0.9	0.7	1.3	1.3	1.4	1.3	0.5	0.9	0.8	1.1	0.4	1.6	0.5	0.6	0.2	0.5	1.6	1.3	0.7	0.6	1.2	1.3	1.2	1.3	1.3	1.3	1.1	1.2	1.1	1.2	1.2	1.2	0.6	0.4	0.7	0.5	0.7	0.9	0.6	0.5	0.6	0.7	0.7
55	0.9	1.5	1.4	1.4	1.4	1.5	0.7	0.9	0.6	1.2	1.2	1.4	1.3	0.4	0.8	0.7	1.0	0.3	1.5	0.4	0.5	0.2	0.4	1.5	1.3	0.7	0.6	1.1	1.3	1.1	1.3	1.3	1.2	1.1	1.1	1.1	1.1	1.1	1.1	0.6	0.3	0.6	0.5	0.7	0.8	0.6	0.5	0.5	0.6	0.7
56	0.7	1.3	1.3	1.2	1.3	1.3	0.7	0.9	0.7	1.0	1.0	1.2	1.1	0.8	1.2	0.8	1.2	0.7	1.4	0.6	0.7	0.6	0.8	1.4	1.1	0.9	0.9	1.0	1.1	1.0	1.1	1.1	1.0	0.9	1.0	0.9	1.0	1.0	1.0	0.5	0.7	0.9	0.6	1.1	1.0	0.7	0.6	0.9	0.8	0.5
57	0.6	1.3	1.2	1.1	1.2	1.3	0.6	0.9	0.6	1.0	1.0	1.1	1.0	0.7	1.1	0.7	1.1	0.7	1.3	0.5	0.6	0.5	0.7	1.3	1.0	0.8	0.8	0.9	1.0	0.9	1.0	1.0	1.0	0.9	0.9	0.9	0.9	0.9	0.9	0.5	0.7	0.9	0.6	1.0	0.9	0.7	0.6	0.9	0.7	0.5
58	0.6	1.3	1.2	1.1	1.2	1.3	0.2	0.4	0.2	1.0	1.0	1.1	1.0	0.7	1.1	0.7	1.1	0.7	1.3	0.5	0.6	0.5	0.7	1.3	1.0	0.8	0.8	0.9	1.0	0.9	1.0	1.0	1.0	0.9	0.9	0.9	0.9	0.9	0.9	0.5	0.7	0.9	0.6	1.0	0.9	0.7	0.6	0.9	0.7	0.5
59	0.9	1.6	1.5	1.5	1.5	1.4	1.0	1.3	1.1	0.7	0.9	0.6	0.5	1.1	1.5	1.1	1.4	1.1	1.7	1.0	1.1	1.0	1.2	1.7	1.3	1.3	1.0	0.2	0.3	0.2	0.3	0.3	0.3	0.2	0.2	0.2	0.2	0.2	0.2	0.8	1.1	1.3	1.0	1.5	1.4	1.1	1.0	1.3	1.2	0.9
60	1.3	0.8	1.1	1.0	1.1	1.7	1.5	1.8	1.8	2.1	2.1	2.2	2.2	1.8	2.2	1.8	2.2	1.8	0.5	1.7	1.8	1.7	1.9	1.8	1.4	1.9	1.8	2.1	2.2	2.1	2.2	2.2	2.1	2.0	2.1	2.0	2.1	2.1	2.1	1.6	1.8	2.0	1.7	2.2	2.1	1.8	1.7	2.0	1.9	1.6
61	1.0	1.3	1.4	1.3	1.3	1.7	1.3	1.6	1.3	1.5	1.5	1.7	1.8	1.4	1.8	1.3	1.8	1.4	1.5	1.3	1.4	1.3	1.5	1.7	1.5	1.5	1.3	1.7	1.8	1.7	1.8	1.8	1.7	1.6	1.7	1.6	1.7	1.7	1.7	1.2	1.4	1.6	1.3	1.8	1.7	1.4	1.3	1.6	1.5	1.2
	1	2	3	4	5	6	7	8	9	10	11	12	13	14	15	16	17	18	19	20	21	22	23	24	25	26	27	28	29	30	31	32	33	34	35	36	37	38	39	40	41	42	43	44	45	46	47	48	49	50

Percent Identity

Divergence

51	52	53	54	55	56	57	58	59	60	61		
99.2	99.3	99.1	99.1	99.2	99.3	99.4	99.4	99.2	98.7	99.0	**1**	MW238796.1
98.7	98.6	98.5	98.5	98.5	98.7	98.8	98.8	98.4	99.2	98.7	**2**	NC_055580.1
98.8	98.7	98.5	98.5	98.6	98.8	98.8	98.8	98.5	98.9	98.6	**3**	MT882412.1
98.8	98.8	98.6	98.6	98.6	98.8	98.9	98.9	98.5	99.0	98.7	**4**	MT882411.1
98.8	98.7	98.5	98.5	98.6	98.8	98.8	98.8	98.5	98.9	98.8	**5**	MT882410.1
98.7	98.6	98.5	98.5	98.5	98.7	98.8	98.8	98.6	98.4	98.3	**6**	MT882344.1
99.3	99.4	99.2	99.2	99.3	99.3	99.4	99.8	99.0	98.5	98.8	**7**	MT311854.1
99.2	99.3	99.1	99.1	99.2	99.1	99.2	99.6	98.7	98.2	98.4	**8**	MT311853.1
99.4	99.5	99.3	99.3	99.4	99.3	99.4	99.8	98.9	98.2	98.7	**9**	MT311852.1
98.9	98.9	98.8	98.8	98.8	99.0	99.0	99.0	99.3	97.9	98.5	**10**	ON470198.1
98.9	98.9	98.8	98.8	98.8	99.0	99.0	99.0	99.2	97.9	98.5	**11**	ON470197.1
98.7	98.8	98.6	98.6	98.6	98.8	98.9	98.9	99.4	97.9	98.4	**12**	ON470196.1
98.8	98.9	98.7	98.7	98.8	98.9	99.0	99.0	99.5	97.9	98.2	**13**	ON937576.1
99.7	99.7	99.5	99.5	99.6	99.2	99.3	99.3	98.9	98.2	98.6	**14**	MZ593773.1
99.3	99.3	99.2	99.2	99.2	98.8	98.9	98.9	98.5	97.9	98.2	**15**	MZ593772.1
99.3	99.4	99.2	99.2	99.3	99.2	99.3	99.3	98.9	98.2	98.8	**16**	MZ593771.1
99.0	99.1	98.9	98.9	99.0	98.8	98.9	98.9	98.6	97.9	98.2	**17**	MZ593770.1
99.7	99.8	99.6	99.6	99.7	99.3	99.3	99.3	98.9	98.2	98.6	**18**	MZ593769.1
98.6	98.6	98.4	98.4	98.5	98.6	98.7	98.7	98.4	99.5	98.5	**19**	OP497960.1
99.7	99.7	99.5	99.5	99.6	99.4	99.5	99.5	99.0	98.4	98.8	**20**	MW759029.1
99.5	99.6	99.4	99.4	99.5	99.3	99.4	99.4	98.9	98.2	98.6	**21**	MW759028.1
99.9	99.9	99.8	99.8	99.8	99.4	99.5	99.5	99.0	98.4	98.8	**22**	MW759027.1
99.7	99.7	99.5	99.5	99.6	99.2	99.3	99.3	98.8	98.1	98.5	**23**	MW759026.1
98.6	98.6	98.4	98.4	98.5	98.6	98.7	98.7	98.4	98.2	98.3	**24**	MW712668.1
98.9	98.9	98.7	98.7	98.8	98.9	99.0	99.0	98.7	98.6	98.5	**25**	MW712667.1
99.4	99.4	99.3	99.3	99.3	99.2	99.2	99.2	98.8	98.1	98.5	**26**	MW988109.1
99.5	99.5	99.4	99.4	99.4	99.2	99.2	99.2	99.0	98.2	98.7	**27**	MW988108.1
98.9	99.0	98.8	98.8	98.9	99.0	99.1	99.1	99.8	98.0	98.4	**28**	MW262984.1
98.8	98.9	98.7	98.7	98.8	98.9	99.0	99.0	99.7	97.9	98.2	**29**	MW262983.1
98.9	99.0	98.8	98.8	98.9	99.0	99.1	99.1	99.8	98.0	98.4	**30**	MW262982.1
98.8	98.9	98.7	98.7	98.8	98.9	99.0	99.0	99.7	97.9	98.2	**31**	MW262981.1
98.8	98.9	98.7	98.7	98.8	98.9	99.0	99.0	99.7	97.9	98.2	**32**	MW262980.1
98.9	98.9	98.8	98.8	98.8	99.0	99.0	99.0	99.7	97.9	98.3	**33**	MW262979.1
99.0	99.0	98.9	98.9	98.9	99.1	99.2	99.2	99.8	98.0	98.4	**34**	MW262978.1
98.9	99.0	98.8	98.8	98.9	99.0	99.1	99.1	99.8	98.0	98.4	**35**	MW262977.1
99.0	99.0	98.9	98.9	98.9	99.1	99.2	99.2	99.8	98.0	98.4	**36**	MW262976.1
98.9	99.0	98.8	98.8	98.9	99.0	99.1	99.1	99.8	98.0	98.4	**37**	MW262975.1
98.9	99.0	98.8	98.8	98.9	99.0	99.1	99.1	99.8	98.0	98.4	**38**	MW262974.1
98.9	99.0	98.8	98.8	98.9	99.0	99.1	99.1	99.8	98.0	98.4	**39**	MW262973.1
99.5	99.5	99.4	99.4	99.4	99.5	99.5	99.5	99.2	98.4	98.8	**40**	MW084633.1
99.7	99.8	99.6	99.6	99.7	99.3	99.3	99.3	98.9	98.2	98.6	**41**	MW600960.1
99.4	99.5	99.3	99.3	99.4	99.1	99.2	99.2	98.7	98.0	98.4	**42**	MW600959.1
99.6	99.7	99.5	99.5	99.5	99.4	99.4	99.4	99.0	98.3	98.7	**43**	MW600958.1
99.4	99.4	99.3	99.3	99.3	98.9	99.0	99.0	98.5	97.9	98.2	**44**	MW600957.1
99.3	99.3	99.2	99.2	99.2	99.0	99.1	99.1	98.6	98.0	98.4	**45**	MW600956.1
99.5	99.5	99.4	99.4	99.4	99.3	99.3	99.3	98.9	98.2	98.6	**46**	MW600955.1
99.6	99.7	99.5	99.5	99.5	99.4	99.4	99.4	99.0	98.3	98.7	**47**	MW600954.1
99.5	99.6	99.4	99.4	99.5	99.1	99.2	99.2	98.7	98.0	98.4	**48**	MW600953.1
99.4	99.5	99.3	99.3	99.4	99.2	99.3	99.3	98.8	98.1	98.5	**49**	MW600952.1
99.4	99.4	99.3	99.3	99.3	99.5	99.5	99.5	99.1	98.4	98.8	**50**	MW600951.1
	99.8	99.7	99.7	99.7	99.3	99.4	99.4	98.9	98.4	98.6	**51**	MW600950.1-
0.2		99.7	99.7	99.8	99.4	99.4	99.4	99.0	98.3	98.7	**52**	MW600949.1
0.3	0.3		99.5	99.6	99.2	99.3	99.3	98.8	98.1	98.5	**53**	MW600948.1
0.3	0.3	0.5		99.6	99.2	99.3	99.3	98.8	98.1	98.5	**54**	MW600947.1
0.3	0.2	0.4	0.4		99.3	99.3	99.3	98.9	98.2	98.6	**55**	MW538943.1
0.7	0.6	0.8	0.8	0.7		99.5	99.5	99.0	98.4	98.8	**56**	MT193106.1-
0.6	0.6	0.7	0.7	0.7	0.5		99.5	99.2	98.4	98.8	**57**	MT193105.1-
0.6	0.6	0.7	0.7	0.7	0.5	0.5		99.1	98.4	98.8	**58**	MT769268.1
1.1	1.0	1.2	1.2	1.1	1.0	0.8	0.9		98.1	98.5	**59**	MT015686.1
1.7	1.7	1.9	1.9	1.8	1.7	1.6	1.6	1.9		98.2	**60**	MT721742.1-
1.4	1.3	1.5	1.5	1.4	1.3	1.2	1.2	1.5	1.8		**61**	SCDZ-Cat
**51**	**52**	**53**	**54**	**55**	**56**	**57**	**58**	**59**	**60**	**61**		
